# Preferential expression of human endogenous retrovirus K (HERV-K/HML-2) type 1 in tumor cells

**DOI:** 10.1186/1742-4690-8-S1-A216

**Published:** 2011-06-06

**Authors:** Camila M Romano, Fernando L de Melo, Paolo M de A Zanotto

**Affiliations:** 1 Department of Infectious and Parasitic Diseases, Institute of Tropical Medicine, School of Medicine, University of São Paulo, São Paulo, SP, 05403-000, Brazil; 2 Laboratory of Molecular Evolution and Bioinformatics, Department of Microbiology, Biomedical Sciences Institute, University of São Paulo, SP, 05508-900, Brazil

## 

Human endogenous retroviruses of the K family (HERV-K) are among the most recently integrated retroviruses in the primate genome. HERV-K(HML-2) is classified in Type 1 that encodes a nonstructural protein Np9 and Type 2 that encodes the accessory protein Rec. Both proteins have been detected in tumors and transformed cell in different levels, and there are evidences that they play a role in cancer development. HERV-K mRNA has also been detected in normal tissues, but its expression is remarkably enhanced in HIV-1 infected patients. Here, we performed an in silico analysis to describe which HERV-K(HML-2) proviruses contribute to transcripts detected in normal tissues, tumor tissues and HIV-1 infected patients. Proviral transcripts from distinct sources (downloaded from GenBank) were subjected to phylogenetic reconstructions together with fifty-five complete HERV-K genomes. Overall, most of the proviruses that did match to transcripts integrated in the host genome between 0 and 7.6 million years ago. A positive relation between proviral activity and integrity of the upstream promoter and GC boxes (putative sites for Sp1 and Sp3 transcription factors) in the 5’ LTR was also noticed. Interesting, while no significant relation was observed between the type of the proviruses and the ability to be transcribed in HIV carriers and normal tissues, 80% of the active proviruses from cancer tissues were type 1 HERV-K. Our results not only describe which proviruses are more active in normal and pathological conditions but also suggest a selective transactivation in tumor cells, supporting the tissue specificity of HERV-K LTR activity.

